# Optimization of BCG Therapy Targeting Neutrophil Extracellular Traps, Autophagy, and miRNAs in Bladder Cancer: Implications for Personalized Medicine

**DOI:** 10.3389/fmed.2021.735590

**Published:** 2021-09-30

**Authors:** Chenyu Mao, Xin Xu, Yongfeng Ding, Nong Xu

**Affiliations:** Department of Medical Oncology Cancer Center, The First Affiliated Hospital, College of Medicine, Zhejiang University, Hangzhou, China

**Keywords:** bladder cancer, Bacillus Calmette Guerin, autophagy, neutrophil extracellular traps, miRNAs, biomarkers

## Abstract

Bladder cancer (BC) is the ninth most common cancer and the thirteenth most common cause of mortality worldwide. Bacillus Calmette Guerin (BCG) instillation is a common treatment option for BC. BCG therapy is associated with the less adversary effects, compared to chemotherapy, radiotherapy, and other conventional treatments. BCG could inhibit the progression and recurrence of BC by triggering apoptosis pathways, arrest cell cycle, autophagy, and neutrophil extracellular traps (NETs) formation. However, BCG therapy is not efficient for metastatic cancer. NETs and autophagy were induced by BCG and help to suppress the growth of tumor cells especially in the primary stages of BC. Activated neutrophils can stimulate autophagy pathway and release NETs in the presence of microbial pathogenesis, inflammatory agents, and tumor cells. Autophagy can also regulate NETs formation and induce production of reactive oxygen species (ROS) and NETs. Moreover, miRNAs are important regulator of gene expression. These small non-coding RNAs are also considered as an essential factor to control the levels of tumor development. However, the interaction between BCG and miRNAs has not been well-understood yet. Therefore, the present study discusses the roles of miRNAs in regulations of autophagy and NETs formation in BCG therapy in the treatment of BC. The roles of autophagy and NETs formation in BC treatment and efficiency of BCG are also discussed.

## Introduction

Bladder cancer (BC) is one of the several types of cancers arising from the tissues of the urinary bladder and is the thirteenth most common cause of mortality and the ninth most common cancer worldwide (1). Conventional factors such as tumor grade, stage, and lymphatic and vascular extension, are utilized as prognostic markers and indicators for BC. However, the currently used prognostic markers have a limited ability to predict progression, recurrence, metastasis, and response to therapy (2). After the initial treatment of BC, a long-term follow-up is essential to prevent BC recurrence. Generally, constant surveillance includes performing a cystoscopy every 3 months for 2 years, then every 6 months for 2 years, and eventually annually, supposing no recurrence (3, 4). Bacillus Calmette Guerin (BCG), as live-attenuated strain of *Mycobacterium bovis*, is considerably similar to *Mycobacterium tuberculosis* in antigenic composition and has been used for treating BC (5). In this respect, immune responses has important role to combat with tumors. Neutrophils are the first leukocytes that counteract against tumor and are able to produce some special compositions that are neutrophil extracellular traps (NETs) (6). Autophagy (autophagocytosis) is defined as the general term for degradation of cytoplasmic components within lysosomes, which is completely different from endocytosis-mediated lysosomal degradation of extracellular and plasma membrane proteins (7–11). Autophagy is classified into three main types including macroautophagy, microautophagy, and chaperone-mediated autophagy and in the medical literature the term “autophagy” is usually referred to macroautophagy unless otherwise specified. A highly specialized organelle called the autophagosome mediates the whole autophagy process through which damaged organelles and cytosolic components are degraded into autophagolysosome, which is created by the fusing autophagosomes with lysosomes (in metazoan cells) or vacuoles (in yeast and plant cells) (12). Autophagy consists of several successive stages mainly including sequestration, transport to lysosomes, degradation, and utilization of degradation products and each of these stages might exert different function. Several studies have shown that BCG therapy can lead to activation of NETs and autophagy, which both prevent tumor growth or metastasis (13, 14). Another effective factor is the epigenetic agents. MicroRNAs (miRNAs) are the non-coding small RNAs that were identified to regulate expression of genes involved in the control of proliferation, development, and apoptosis (15). Additionally, findings of several animal model and human studies have indicated that miRNAs might contribute in suppressing the growth of tumor cells in a manner that imbalance of miRNAs gene expression could result in excessive proliferation of cancerous cells. In this regard, miRNAs play a crucial role in prognosis of the BC especially initial phase (16). Furthermore, miRNAs have also capacity to stimulate autophagy and NETs formation in neutrophils and inhibit tumor metastasis (17). BCG therapy could prevent recurrence and progression of tumor in BC. However, dysregulation of some cellular and molecular processes such as autophagy pathway and NETs formation could result in metastasis stimulation in BC (18). Dysregulation of miRNAs expression facilitates the growth and proliferation of tumor cells and tumor invasiveness can be promoted by autophagy process and NETs formation (19). Interestingly, therapeutic options may serve as regulatory agents to inhibit cancer progression and improve BCG therapy efficacy through complex network of miRNAs, autophagy and NETs, which can have significant effects on the efficacy of BCG therapy in BC treatment (20–22). Although BCG therapy has been administrated for BC treatment for decades, its therapeutic efficacy should be more evaluated to elucidate the roles of miRNAs in autophagy regulation and NETs formation and their mutual interactions. Moreover, miRNAs are important regulator of gene expression. These small non-coding RNAs have been reportedly considered as pivotal factors controlling and regulating the levels of tumor development. However, the interactions between BCG and miRNAs have not been well-understood yet. Therefore, the present study discusses the roles of miRNAs in regulations of autophagy and NETs formation in BCG therapy in the treatment of BC. The roles of autophagy and NETs formation in BC treatment and efficiency of BCG are also discussed.

## BCG Therapy In Bladder Cancer

Morales et al. was the first group reported the treatment efficacy of BCG therapy for BC (23). After several clinical trials and strong evidence on the efficacy of this technique, intravesical BCG has been established as a standard treatment for high-risk, non-muscle-invasive BC in different stages including lamina propria-invasive tumors (stage T1), carcinoma *in situ* (CIS) (stage Tis), and high-grade papillary tumors (stage Ta) (24, 25).

In these situations, BCG therapy could be correlated with a decreased risk of recurrence compared with transurethral resection alone, and the risk of progression to invasive disease would also be reduced by using BCG therapeutic approach (26–28). In addition, findings of the recent studies have demonstrated that the effectiveness and therapeutic outcome of BCG therapy in BC are comparable with intravesical chemotherapy, meanwhile BCG therapy is more effective in decreasing the risk of tumor recurrence, but the its toxicity can be more severe (29–31).

## Neutrophils and Cancer

Neutrophils as a crucial element of innate immunity in any organisms play important roles in responding to different inflammatory and invading pathogens such as microbes, bacteria and fungi (32). These predominant leukocytes are among the first blood cells recruited to an inflammatory site (33). NETs are a network of chromatin structure with related enzyme including elastase, myeloperoxidase, and cathepsin G which were released by stimulation with phorbol myristate acetate (PMA), carcinogenesis substance. NETs can trap, neutralize, and kill the extracellular bacteria, viruses, fungi, and parasites. Moreover, NET release occurs initially through a cell death process termed NETosis (34). This process begins with interrupt of the nuclear envelope and continue with chromatin decondensation into the cytoplasm of intact cells. Moreover, NETiosis can occur following the secreted nuclear chromatin that is accompanied by the release of granule proteins through degranulation (35).

Neutrophils have been reportedly to involve in different biological functions including phagocytosis, secretion of chemo-attractant and degranulation, and respiratory burst. Recent evidence has demonstrated a new biological function for neutrophils that is releasing of NETs (34, 36). NETs are specialized network structures composing mainly of histones, de-condensed chromatin, and effector cytokines, that is, myeloperoxidase (MPO). The main strategy of neutrophils for triggering immune defense response to prevent the invading pathogenic microorganisms from escaping the immune system and expanding the infection, neutrophils first locate and capture the pathogens through releasing NETs and subsequently trigger other immune cells and initiate systemic immune defense.

## NETosis and Cancer

The role of NETs in tumor progression remains poorly understood. The findings of the both animal and human studies suggest a potential association between tumor progression and intra-tumoral NET deposition in both experimental models and in human cancer patients (36–38). Zychlinsky et al. evaluated the presence of tumor-associated neutrophils (TANs) and NETs in surgical resection specimens from eight patients with sarcoma as determined by positive staining for extracellular myeloperoxidase (MPO) 25% of these patients (2 patients), demonstrated intra-tumor NET deposition (36). These two patients developed early relapse after performing post-neoadjuvant chemotherapy and surgery, although the site was not specified in the study (36). Therefore, it seems that Ewing sarcoma cells can stimulate TANs to release NETs. The ability of tumor cells to involve neutrophils to produce NETs has been displayed in a number of tumor types. This phenomenon indicates the possibility that NETs play a fundamental role in tumor biology (39). In this regard, Demers et al. demonstrated that several tumor types including lung neoplasms and mammary, hematologic are able to involve circulating neutrophils to produce NETs (39). The evidence presented in the literature thus far suggest that NETs my promote tumor progression within the primary tumor (40).

As previously stated, NETs have usually strong adhesive characteristics, which enable them to bind pathogens and platelets. It, thereby, seems to hypothesis that NETs also provide intravascular networks facilitating tumor cell adhesion and extravasation in hematogenous metastasis. Actually, neutrophils is able to promote the arrest of circulating tumor cells, especially under inflammatory conditions, which remarks at a role of NETs in this process (41–43). Additional direct evidence arises from a recent *in vitro* study demonstrating that lung carcinoma cells display 4–5 fold increased adhesion to NETs as compared with unstimulated neutrophil monolayers (37, 44).

Another important aspect which should be paid attention is the role of neutrophil in the cancer microenvironment. In this regard, some studies which has been recently performed in the field of neutrophil roles in tumor microenvironment suggest that neutrophil exhibit substantial plasticity which could be polarized to an N1 antitumoral or N2 protumoral phenotype in response to the microenvironment, the same of the M1/M2 macrophages polarization (45, 46). Tumor-associated N2 neutrophils are identified by high expression of VEGF, CXCR4, gelatinase B, and MMP9 and can be induced on exposure to high TGF-β levels. Vice versa, N1 neutrophils express immunopotentiating cytokines and chemokines such as IFN-γ, CXCR3, and low levels of arginase and also are induced on TGF- β blockade and are able to eliminate cancer cells (45, 47).

In this regard, a performed study by Berger-Achituv et al. showed that NETs have either pro– or anti-tumor function, depending on factors such as tumor microenvironment and type of cancer. For instance, within the microenvironment of the tumor, TGF-β can induce TANs with pro-tumorigenic features. However, TANs produce pro-inflammatory cytokines and have tumoricidal activity without TGF-β (45).

In addition, neutrophils can enhance tumor growth through production of matrix metalloproteinase (MMP)-9 that inhibits tumor cell apoptosis in the respiratory tract and can increase tumor angiogenesis and neovascularization (48, 49). Nevertheless, neutrophils can also have cytotoxic effects on tumor cells by generating many types of reactive oxygen species (ROS) (50, 51). Notably, in a study was demonstrated that neutrophils inhibited metastatic seeding by secreting hydrogen peroxide in a mouse model of breast cancer (52). Neutrophils also secret defensins, which have anti-angiogenetic characteristics and can lyse cancer cells, recruit dendritic cells (DCs) as antigen presenting cell (53). NETs are thought to have anti-tumorigenic effects, for example through activating immune responses and killing of tumor cells. On the other hand, NETs could have a pro-tumorigenic function by promoting metastases. In fact, NETs may act to physically take tumor cells and inhibit their dissemination to adjacent tissues. Various components of NETs have been indicated to be cytotoxic to tumor cells. MPO was demonstrated to destroy B-16 melanoma cells and prevent their growth in mice after implantation (54). Interestingly, patients with MPO deficiency probably have a high incidence of cancer (7/14 patients, 50%) (55). NETs can eradicate activated endothelial cell, may by histones, damaging tumor-feeding blood vessels (56). NE produced by TANs cleaves Cyclin E to other isoforms with lower molecular weight and therefore facilitates their presentation to cytotoxic T cells (CTLs) (57). Indeed, NETs have a modulatory role to establish the bridge between innate and adaptive immunity by activating plasmacytoid DCs through toll-like receptor 9 (TLR9), an intracellular receptor that preferentially binds DNA. NETs have capability to prime T cells by TCR signaling that implicates direct contact (58). Alternatively, NETs, which contain different proteases, could represent a pro-tumorigenic activity by degradation of the extracellular matrix and increase metastasis. NETs may also create a hurdle between cancer cells and the immune system, thereby collaborating with cancer cells to evade from immune recognition. Consequently, it has been reported that patients with metastatic disease showed NETs formation relapsed that may refer to the pro-tumorigenic mechanism of NETs (36). Moreover, there is a recent evidence indicating that neutrophils from certain donors have capable to kill cancer cells in a cell-specific manner and that neutrophil killing of cancer cells may be improved by β-glucan treatment, making neutrophil a persuadable candidate for cancer immunotherapy (59). Various studies that induce neutrophilia through prolonged G-CSF treatment in tumors show a shift form a chronic to an acute inflammatory environment and an anticancer effect (60). Notably, mammary tumor cell lines stimulate NETosis *in vitro*, but there is no strong evidence for NET formation in these tumors *in vivo* (61). On the other hand, some studies have been demonstrated that NETiosis is able to counteract against cancer metastasis (47). One underlying mechanism for metastasis suppression seems to be the NET-mediated capture of migrating tumor cells, particularly at places of inflammation, which can be blocked with neutrophil elastase (NE) and protein-arginine deiminase type 4 (PAD4) inhibitors (62). Therefore, targeting NETs through these pathways could be a promising therapeutic option to treat cancer. In the next section we will discuss about the role of BCG therapy in BC and the interaction between NETs and BCG for treatment of BC (62).

Collectively, neutrophil-induced NETs act as an inhibitor for development of tumor metastasis through elastase, MPO, and other enzymes. In contrast, a few evidence demonstrated that produced NETs from the TANs in microenvironment of tumor could lead to progress of tumor cells.

## Neutrophils In BCG Therapy

BCG instillation into bladder provides a localized infection that involves both attachment and then internalization into normal and malignancy urothelial cells through fibronectin process mediated by integrin adhesion molecules (63–65). Recent studies have demonstrated that neutrophils are able to migrate to the bladder after BCG stimulates bladder epithelial cells to secrete chemokines. Also, another study showed that neutrophils have important role in anticancer outcome of BCG therapy. In this regard, Suttman et al. reported that neutrophils could be reason of positive outcome to BCG therapy in a mouse bladder tumor model (66). They found that BCG therapy has no effect after depletion of neutrophil that result in a reduction in survival compared with non-depleted controls. Neutrophils release IL-8, MIF, MIP-1α, and GRO-α when are stimulated with BCG *in vitro*. Therefore, BCG-induced chemokine secretion by neutrophils is sufficient to recruit macrophages, which eventually recruit T cells. According to these findings, Suttman and their colleagues, suggest that BCG administration can result in the influx of neutrophils that coordinate the subsequent macrophages and T cell recruitment via the release of chemokines (66). Interestingly, in consideration of Suttman et al.'s results, it is proposed that the BCG-induced antitumor responses are mediated by activated T cells, whereas neutrophils recruit other immune cells with indirectly mechanism (65, 66). Additionally, neutrophils have a direct antitumor immunity through the production of soluble TRAIL, tumor necrosis factor related apoptosis inducing ligand, into the bladder environment (65).

In another study, Liu et al. evaluated the formation of NETs by induction of BCG instillation. They have shown that tumor cell proliferation was inhibited by treatment with NETs as well as cytotoxicity of NETs on tumor cells. Their results demonstrated that BCG-induced NETs promote dose-and time-dependent apoptosis of tumor cells and G0/G1 phase arrest. Obtained findings from the Liu et al.'s study demonstrated BCG-activated tumors stimulated more NETs than non-activated ones. Also, neutrophil adhesion and NETs release were increased by stimulation with supernatant of activated cells which representing a significant role for cytokines. Their results also suggest that BC cells induce NETs via TNF-α and IL-8 secretion following BCG stimulation. Eventually, they concluded that BCG-induced NETs suppressed tumors through multiple mechanisms including cytotoxicity effects, induction of apoptosis and cell cycle arrest. Some studies have been shown that NETs could suppress migration and invasion of tumor cells by induction of apoptosis and exert cytotoxicity mechanism on tumor cells (13). Besides, role of Neutrophils has been proven for T cells trafficking to the bladder after BCG perfusion (66). Actually, CD4+ T cells are the main contributors in BCG therapy, according to IFN-γ cytokine production or activation of CD8+ T and NK cells (66, 67). Furthermore, NETs could prime T cells and activate dendritic cells (DCs). NETs treatment upregulate CD4 expression *in vitro*, and CD3+ and CD14+ cells in tumors that could be an important index for potentiating of immunity. Generally, the presence of monocyte, Th1 cells, and CTLs in environment of tumor could result in tumor regression and also this cellular population can coincide with a favorable prognosis (13). It has been demonstrated that NETs have either tumor pro- or anti-tumor activity. Therefore, some agents such as cytokine profile in the microenvironment and cancer type are the determinative subject for progression or suppression of tumor cells (68, 69). At high concentrations, release of MPO and NE as important components of NETs have cytotoxicity effect on tumor cells, but reducing the release may result in the conversion of anti-tumor to pro-tumor function (70, 71). Therefore, NETs have different roles based on variation in multiple stimuli, neutrophil action site, and induction with BCG or others (13).

Collectively, neutrophils are induced by BCG activation to form NETs. In other words, the direct role of BCG-induced NETs has been indicated by cytotoxicity effect, apoptosis induction, cell cycle arrest, and inhibition of tumor cells migration into bladder environment. Besides, NETs have also indirect role through stimulation immunity and recruitment of T cells and macrophages to prevent tumor growth. In the next section, it will be discussed about the effect of autophagy pathways and its relation with NETs and BCG therapy in BC.

## Autophagy and NETs

The autophagy functions can be classified into two categories including generation of essential metabolic degradation products and clearance of intracellular defective organelles and macromolecules (72).

One of the best conserved-function of autophagy is associated with adaptation to starvation among many various organisms. When growth requirements increase or nutrients are scarce, metabolic intermediates were produced by autophagy contribution which mainly happened for sustaining cell survival (73). The mTOR as a serine/threonine protein kinase can form two different protein complexes that known as mTORC1 and mTORC2. Evidence shows that mTOR is a main control way for autophagy pathway, which mTORC1 could control catabolic activity via the process of autophagy. This mechanism is proceed by integrates signals from multiple pathways, sensing the levels of nutrients and growth factors (74). Along with, AMP-dependent protein kinase (AMPK) is the key sensor of cellular energy status which activate autophagy pathway (20–25). Substantial regulators of autophagy are the class I and class III phosphatidylinositol 3-kinase (PI3K) pathways. Class I PI3K activates mTORC1 and inhibits the beginning of autophagy. In contrary, class III PI3K can induce autophagy directly (75).

Initiation, elongation, autophagosome completion, fusion with the lysosome, and degradation are five steps of autophagy process (76). When nutrients and energy in cells are empty, mTORC1 is inactivated, it can no longer prevent the autophagy initiation complex which are involving the protein kinases unc-51-like kinases 1 and 2 (ULK1/2), ATG13, ATG101, and FIP200. These protein kinases are able to form a newly phagophore membrane carrying ATG14, endoplasmic reticulum-associated protein. Recruitment of Beclin 1, Vps34, and Vps15, was performed through the nascent phagophore membrane which in turn results in the formation of an activated class III PI3K complex that also produces phosphatidylinositol 3-phosphate (PI3P) (77). The elongation step initiates the enlargement and final closure of and extending membrane, leading a completed autophagosome. Double-membrane organelle would be formed by two ubiquitin-like protein-conjugation systems, essential for the elongation phase. Dissociation of ATG12-ATG5 conjugation occur from the outer autophagosomal membrane after that vesicle formation is complete. Microtubule-associated proteins 1A/1B light chains (LC3) is another conjugation system which are cleaved by ATG4 upon autophagy induction, causing cytoplasmic LC3-I. In addition, LC3II conjugation complex was created through more lipidation with phosphatidylethanolamine (PE), which is then combined into both inner and outer autophagosomal membranes. The presence of LC3-II in the autophagosomal membranes is commonly considered as a marker for detection of double-membrane autophagic organelles. On the other hand, p62 is necessary for aggregating and binding polyubiquitinated protein to LC3-II to provide a situation that phagophore could engulf cytosolic elements, to grow, and consequently to close the autophagosome. Generally, accumulation of p62 occurs when autophagy is inhibited, and the decrease of p62 also represents suitable vesicle degradation and autophagic flux (78–80). At the final steps, the autophagolysosome content, which is produced by the fusion of autophagosome with lysosome is degraded via hydrolytic enzymes (18, 76, 81, 82). In this regard, the role of autophagy is important for major neutrophil functions, including phagocytosis, differentiation, degranulation, cytokine production, cell death, and NETs formation. ATG proteins are main members in the neutrophil differentiation pathway. ATG5 is needed in both canonical and non-canonical autophagy. In addition, the role of ATG5 has been demonstrated to differentiate neutrophil (83). Moreover, mTORC1 has a pivotal role in the regulation of autophagy so that differentiation of neutrophilic precursor cells could be ceased by using pharmacological inhibition of mTORC1- induced autophagy – or p38 mitogen-activated protein kinase (MAPK) (77). Therefore, autophagy exhibits a mutual regulating interaction by the p38–mTORC1 axis (83).

Different *in vivo* studies have demonstrated that metabolism and autophagy are programmed for neutrophil differentiation. In this regard, findings of some studies have exhibited that reduced ATG gene expression is correlated with acute myeloid leukemia (AML) samples (84). It has been demonstrated that neutrophils were primed by autophagy for increased NETs formation, which is notable for appropriate neutrophil effector functions during sepsis (85). Actually, neutrophils have a potency to increase autophagy induction in patients who survived sepsis. On the other hand, there is an abnormally autophagy function in neutrophils isolated from patients who could not survive sepsis. However, in murine models of sepsis, the autophagy reinforcement improved survival through a NET-dependent mechanism (86).

Interestingly, autophagy and ROS production as two main regulators of NETosis have a close correlation to each other. Autophagy induction can occur through ROS burst, which in turn is necessary to maintain effective ROS production (87).

**Figure 1:**
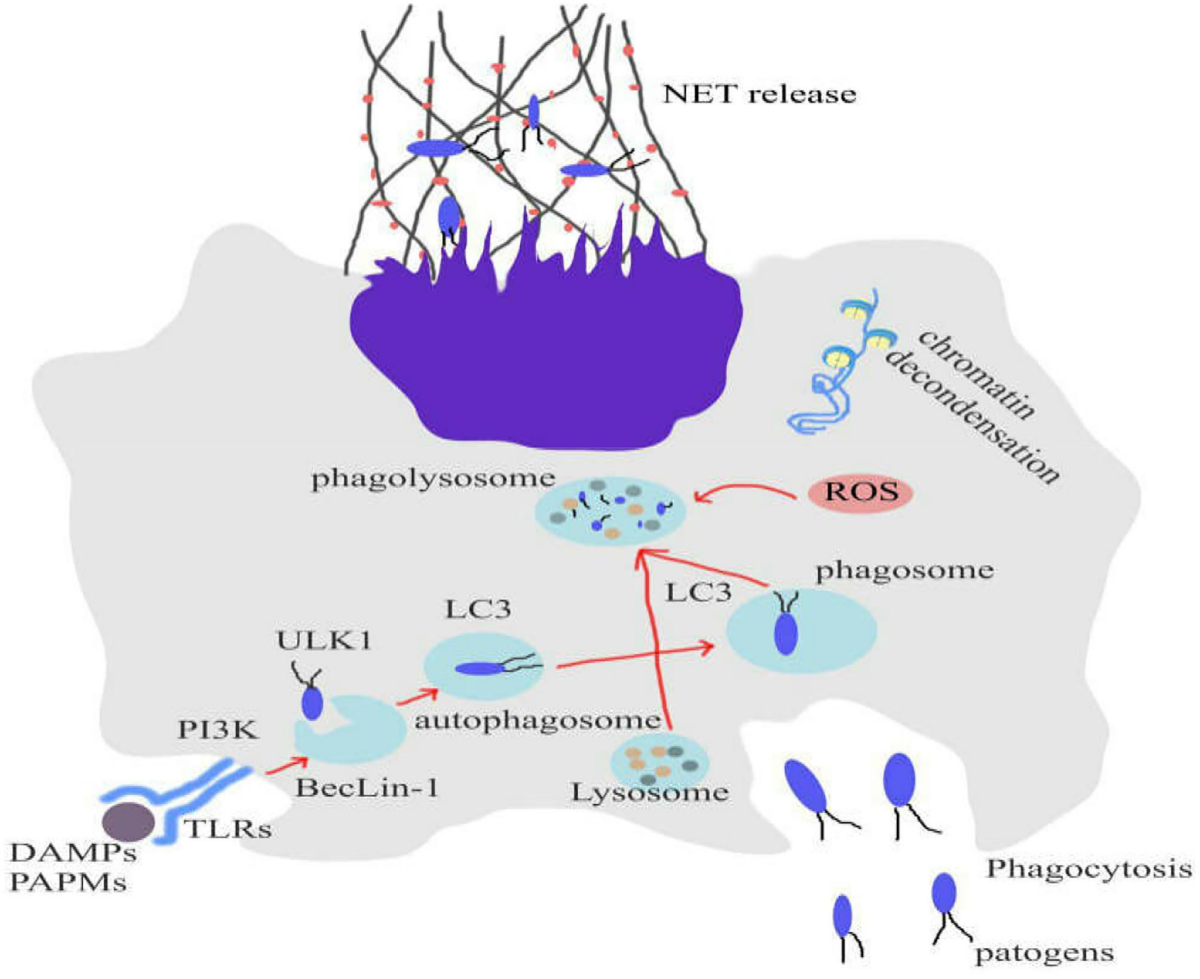
Role of autophagy and ROS production to form NETosis. Inhibition of either NADPH oxidase or autophagy could prevent the chromatin decondensation that is crucial for NETosis, resulting in apoptotic cell death. NETosis can eliminate infections and tumors.

Remijsen et al. have studied the roles of autophagy and ROS production in formation process of NETosis (88). Their findings demonstrated that a combination of ROS production and autophagy is required for PMA-induced-NET formation in human neutrophils. Inhibition of either NADPH oxidase or autophagy could prevent the chromatin decondensation that is crucial for NETosis, resulting in apoptotic cell death. Additionally, they reported that there was not any NADPH oxidase activity in neutrophils, isolated from patients with chronic granulomatous disease (CGD). The evidences showed that these neutrophils are incapable of producing NETs (88) (Figure 1).

Similarly, other studies have demonstrated that neutrophils from patients with acute gouty arthritis (AGH) display autophagy-mediated spontaneous NET release (89). Currently, it has also been demonstrated that reduced expression levels of Atg5 interplayed to decreased capacity of neutrophils to form NETs when TLR2 ligand stimulation has occurred in aged mice. This suggests that it may represents a major role of autophagy in maintaining the mechanism of NETs (90).

On the other hand, some studies have reported contradictory findings on the contribution of autophagy in NET release. Particularly, *Atg5*-knockout mouse neutrophils, that display decreased autophagic activity, kept the capacity to release extracellular DNA. Moreover, PI3K is able to prevent NET formation inhibition by human neutrophils (91). Consequently, it maybe exists an autophagy-independent NETosis pathway (91, 92).

Collectively, activated neutrophil may promote autophagic activity and NET formation. Also, autophagy induces NET formation. But in relation to cancers specially BC, whether autophagy is able to induce NETs formation has yet to be determined and now there are no papers which discussed clearly in the literature. However, according to similar studies which have been conducted, it seems that autophagy-induced NETs formation would happen in tumor microenvironment through tumor-associated neutrophils (N2). However, further investigations certainly should be performed to clarify the matter.

In the next section, the role of autophagy in cancer and angiogenesis will be discussed.

## Autophagy and Cancer

Findings of the studies on the role of autophagy process as a driver of cell death or a pro-survival process in response to specific stressors are controversial. Autophagy has been initially described as a cytoprotective process under nutrient deprivation, whereas recent findings of several studies have demonstrated that autophagy process is a cell death driver in which it is involved in promoting cell death.

Recently, the paradoxical role of autophagy in cancer progression or suppression has been widely evaluated. Actually, cancer type, genetic context, and stage were affected by autophagy which can determine tumor cell destiny (93). Some studies demonstrated that autophagy is established as a tumor suppressive mechanism and the autophagy defective could be related to genomic instability, malignant transformation, and tumorigenesis (94, 95). Also, Beclin-1 acts as a tumor suppressive that allelic loss of this gene can results in incidence of some types of cancer including prostate, ovarian, and breast cancer (41, 94, 96). Moreover, the tumor suppressor function of Beclin-1 is exerted through binding and activating Vps34 which lead to induce autophagy (97). In this regard, Autophagy is able to maintain genome integrity and inhibit tumor initiation. Deletion of tumor suppressor PTEN and elevation of the PI3K/Akt/mTOR pathway, which is prevalent in many cancers, could be the cause for reduced cytoprotective autophagy and uncontrolled proliferation (98). Evidence shows that mTOR signaling suppresses the pro-autophagic protein AMBRA1, which can regulate cell proliferation by dephosphorylating c-myc (99). These Interpretations imply that impairment of autophagy can increase the risk of tumors (96, 100). Meanwhile, the case of established tumors is completely wrapped and the modulating role of autophagy in cell proliferation is highly context-dependent. High levels of autophagy are often occurred in cancers with BRAF and KRAS driver mutations. This increased autophagy is crucial for PDAC tumor growth and sustenance, and halting it results in tumor regression (101). Similarly, Atg7 deletion in BRAF-driven lung cancer model cause to inhibit autophagy pathway that indicating tumor regression and reversal of malignancy (102). There are various opposite interpretations that challenge the proliferative roles of increase autophagy in tumor cells. For example, Results of studies suggest autophagy inducers including rapamycin and its derivatives which are known inhibitors of mTOR, can also prevent mTOR-dependent cell proliferation via induction of cell cycle arrest in MDA-MB-231 breast cancer cells and cell lymphoma (103, 104).

In conclusion, the evidence demonstrates that autophagy can regulate proliferation in a context-dependent manner. These studies express coordination of autophagy with proliferation that support a dual function of autophagy in one of the essential indexes of cancer.

## Autophagy and Angiogenesis

Tumor angiogenesis occur with formation new blood vessels from the existing vasculature. To angiogenesis, tumor needs to some growth factors such as vascular endothelial growth factor (VEGF) and tumor growth factor-β (TGF-β). Angiogenesis supports tumor growth by providing nutrients for cancer cells, consequently aiding in tumor growth, invasion, and metastasis (105, 106). Additionally, when vascular supply of cells is terminated, the hypoxic situation is established and autophagy induced through HIF-α-mediated signaling (107). The increased levels of autophagy can facilitate tumor cells survive to sustain oxygen stress and could become resistant without blood supply. On the other hand, a specific role of autophagy in angiogenesis has been reported in neuroblastoma cells that showed that autophagy is able to suppress angiogenesis through degradation of pro-angiogenesis peptide which is called gastrin-releasing peptide (GRP) (108). Autophagy inhibits tumor cell necrosis and inflammation and mediates nutrients and hypoxia. It therefore diminishes the recruitment of macrophages at the primary tumor site, which is important for metastasis induction. Inhibition of epithelial-mesenchymal transition (EMT) by autophagy could occur through degradation of p62/SQSTM1 and its cargo TWIST, which promotes EMT. TWIST is helix-loop-helix transcription factor that regulates human osteogenic linage (109).

The process of migration and metastasis initiates when the cells lose contact with adjacent cells, detach from extracellular matrix (ECM), undergo EMT, and eventually become motile. Anoikis, as a type of apoptosis, occurs after tumor cells detach from surrounding ECM. However, tumor cells can evade from anoikis via autophagy induction that provides Anoikis resistance (110). When the separated tumor cells reach the favorable site, they may remain latent until they can find new contacts with the ECM. At this stage, autophagy generally helps in their survival through unknown mechanisms. For instance, ARH1, tumor suppressor gene, is able to induce autophagy and increase tumor cell latency in ovarian cancer. Latency tumor is a barrier for cancer treatment (109). Moreover, studies have determined that autophagy induction by starvation results in promoted metastasis and invasion of hepatocellular carcinoma cells. This event regulated by TGFβ/smad3 signaling (111).

Collectively, autophagy can suppress incidence of invasion and metastasis by inhibiting inflammation and tissue necrosis. But if the tumor cells detach from ECM, elevated levels of autophagy help them avoid apoptotic cell death and maintain latency in a distant site (109, 110). Thereby, autophagy acts as a double-edged sword in tumor progression or suppression (112).

## Autophagy and BCG

In this regard, it has been demonstrated that there is a relationship between BCG therapy and autophagy pathway. Because both BCG and wild-type *Mycobacterium tuberculosis* secrete many antigens including the ag85 complex, we concluded that generated antigens could be targeted into the autophagic pathway. It seems that such an event would promote the production of peptides from ag85 complex and their subsequent loading on to MHC II proteins. A study evaluated the effect of induction autophagy on the efficacy of BCG vaccine containing the immunodominant Ag85B. Their results showed that induction of autophagy increases Ag85B presentation through MHCII pathway and thereby elevates vaccine efficacy. Also, autophagy increases Ag85B presentation by macrophages (113).

In a comparative study evaluated the effect of BCG on gastric cancer cell line MGC-803. They reported that BCG therapy increases protein level of Atg-3 and lymphocyte immunocompetence to induce cell apoptosis and autophagy in gastric cancer cells (114). Collectively, some studies indicated that BCG is tightly associated with induction of autophagy in various cancers. BCG therapy is able to induce autophagy pathway and kill the tumor cells. Therefore, autophagy can be considered as an important agent to reinforce the effect of BCG on inhibition of tumor growth.

Recently, it has been demonstrated that there is an association between protective mechanisms of BCG and epigenetic alternations in innate immune cells (115). In this regard, Buffen et al. have studied the effects of BCG therapy on autophagy and its relation with epigenetic alternation in BC. Their findings demonstrated that BCG-induced autophagy could act as a central event modulating epigenetic alternations on innate immunity. Furthermore, they reported polymorphism in ATG2B gene controls epigenetic alterations in both *in vivo* and *in vitro* models in BC (20). Thereby, epigenetic alterations are the noteworthy topic in BCG therapy. In this respect, epigenetic and miRNAs are regulators of gene expression. The current literature shows that miRNAs play a crucial role in autophagy pathway, BCG therapy and NETs process. We will discuss these roles in next sections in more details.

## MiRNAs Role In Autophagy and BCG Therapy

MiRNAs is a group of non-coding small RNAs that can have different effects on oncogenesis by acting as oncogene or tumor suppressor in microenvironment-dependent manner (116).

Various studies conducted on the roles of miRNAs in BC have demonstrated that some miRNAs are overexpressed, whereas some other miRNAs are down-regulated during BC development. Gottardo et al. evaluated expression of miRNAs in 27 bladder specimens. They found that miR-17-5p, miR-23a, miR-23b, miR-26b, miR-103-1, miR-185, miR-203, miR-205, miR-221, and miR-223 were remarkably upregulated in BC. In fact, the function of a specific miRNA depends on its target genes. Thus, upregulated miRNAs that impact oncogenes can be considered as tumor-suppressing miRNAs, and downregulated miRNAs that impact tumor suppressor genes can be considered as onco-miRNAs (117). Another study showed that expression of four miRNAs (miR-199a-3p, miR-195, miR-133a, and miR-30a-3p) was downregulated in tumors, however, these miRNAs usually act as tumor suppressors (37). On the contrary, miR-200c and miR21 are upregulated in BC tissues and could be an agent for the progression of BC (118, 119). The expression of miRNAs are detected using molecular techniques such as microarray or deep sequencing in patients with BC. Many types of samples obtained from clinical tissue specimens, fluids, body, and BC cell lines (120–122). Because autophagy incidence is inside cells, the present study evaluates the expression of miRNAs in BC tissue samples. miRNAs have several roles in the regulation of autophagy processes such as recycling, degradation, fusion, vesicle nucleation, vesicle elongation, and induction (123).

As mentioned in previous section about autophagy process, the ATG13, FIP200, and ULK1/2 and its negative regulator mTORC1 are the components of ULK complex which are required for initiating the autophagy process. It has been reported that the miR106b and miR20a have a repress activity on autophagy by targeting ULK1 (124). Another study demonstrated that the miR25 has a direct effect on ULK1 expression and it was considered as a novel regulator of autophagy (125). Moreover, miR26b can inhibit autophagy by targeting ULK-2 (126). In addition, miR20a has been reported to modulate autophagy through targeting FIP200 (127). MiR15a, miR16, and miR18 exert pro-autophagic effect by inhibiting mTORC expression and have been recognized as onco-miR (128, 129). The Beclin-1-PI3KCIII-Vps15 complex, ATG2-18 complex, and ATG9 are fundamental components for the vesicle nucleation as the second step of autophagy process. Several miRNAs can regulate this step of autophagy. For instance, miR30a AND miR17 were identified to suppress Beclin-1 expression, therefore disrupting vesicle nucleation (130, 131). Also, the results of some studies have been revealed that miR199a and miR152 can directly target ATG14 to negatively regulate the activation of the Beclin-1-PI3KCIII-Vps15 complex (132, 133). Furthermore, studies have reported that activity of ATG2-18 complex and ATG9 are repressed by miR130a and miR34a (134, 135). Several miRNAs contribute in regulating the expression of ATG12-5-16 components such as miR30a, miR23b, and miR106b throughout the process of vesicle elongation and completion (125, 136, 137). Moreover, there are other reports indicating that miR101 can target ATG4 to suppress autophagy (138). In addition, miR199a and miR423 have been reported to regulate the resistance by targeting ATG7 and develop autophagy (116, 139). MiR204 was demonstrated to stop the activation of ILC3-II, applying a similar effect in this process (140).

**Figure 2:**
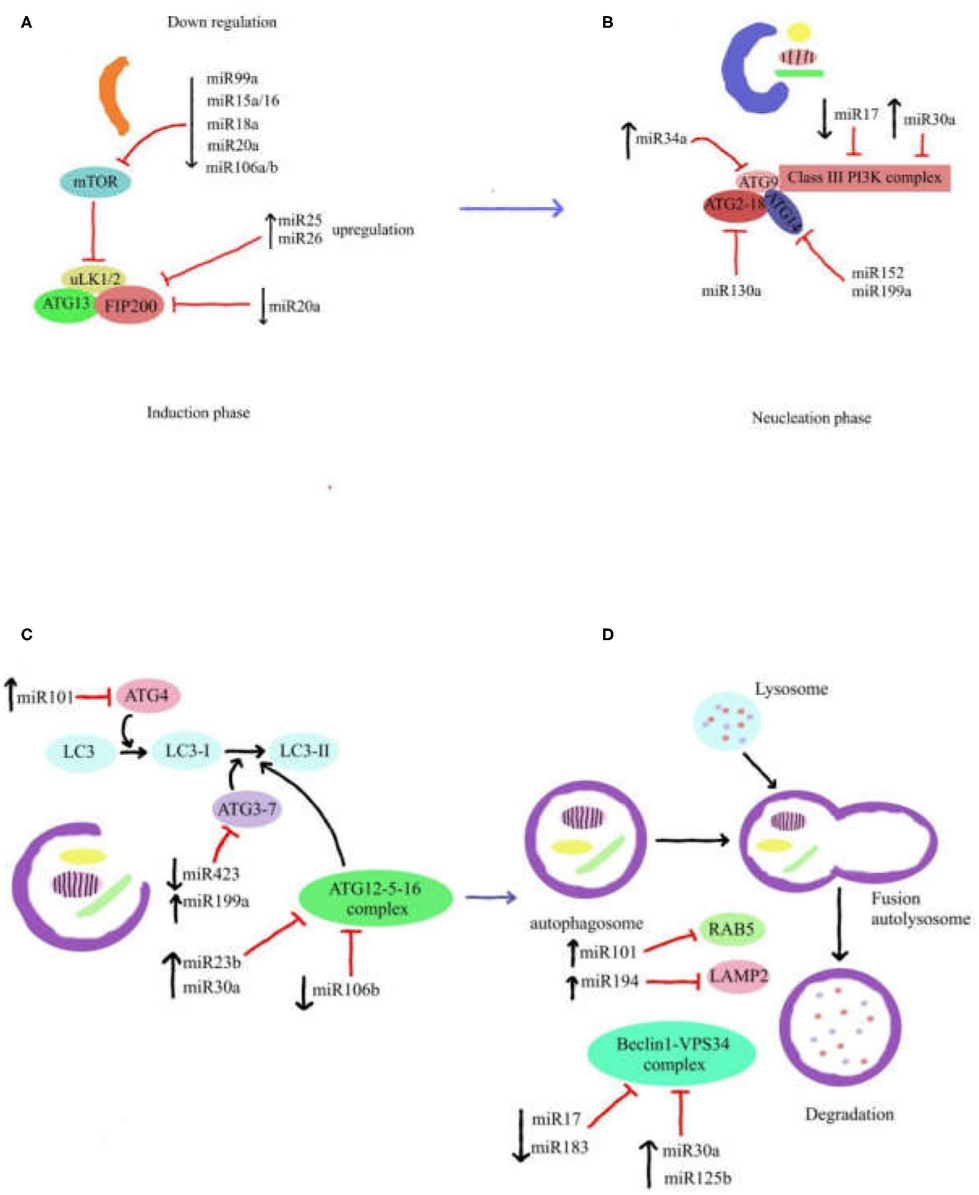
Role of miRNAs in autophagy pathway. Autophagy process is illustrated in four steps **(A–D)**, in which miRNAs affect each of autophagy components. The various roles of miRNAs demonstrate that miRNA gene expression can regulate components of autophagy pathway. Some of these miRNAs have important role in each step of autophagy process. Autophagy pathway can be also inhibited by downregulation and/or upregulation of miRNAs gene expression.

Eventually, the cargo inside and autolysosome maturation undergo degradation and recycling process. MiR101 was identified to inhibit RAB5, a main regulator of autolysosome fusion (138). In addition, a study reported that miR194 has an essential role in autolysosome fusion through targeting LAMP2 (141). UV Radiation Resistance Associated Gene (UVRAG) which is a main component of the Beclin1-Vps34 complex and plays a central role in autolysosome maturation. miR-183 and miR-125b were discovered to target UVRAG (142, 143) (Figure 2). ATG7 is overexpressed in human invasive BC tissues. It has been demonstrated that miR190a facilitates BC invasion and autophagy through stabilizing ATG7 mRNA by binding to its 3′UTR (144).

Collectively, dysregulation of miRNAs serves to progress the cancer through targeting components of autophagy pathway. Thereby, modification of miRNAs expression could help to suppress the invasion of cancer.

As discussed above, the main objective of BCG therapy is preventing occurrence and progression of cancer. Some studies have indicated that MImiR-9-3, miR-124-2, miR-124-3, and miR-137 were frequently methylated in the initiation phase of cancers which can be utilized as potential biomarkers for BC diagnosis (145). Interestingly, to our knowledge there is no published study that had investigated the possible effects of BCG on miRNAs profile expression. However, several studies have been conducted on the roles of BCG and miRNAs in infectious diseases. For examples, a performed study evaluated the alteration of immune-related miR142-3p in macrophage RAW264.7 cells in treatment with BCG infection (146). Their results showed that miR142-3p can negatively regulate the production of pro-inflammatory mediators IL-6, TNF-α, and NF-κB (NF-κB1) in the macrophages by post-transcriptionally down-regulating IRAK-1 protein expression (146). Moreover, another study demonstrated that *M. bovis* BCG induces Toll-like receptor 2 (TLR2)-dependent miR155 expression, which establishes signaling cross talk among mitogen-activated protein kinases (MAPKs), protein kinase Cδ (PKCδ), and phosphatidylinositol 3-kinase (PI3K) and recruitment of c-ETS and NF-κB to miR-155 promoter. Finally, they indicated the cellular reprogramming was organized by miR155 during immune responses to mycobacterial infection (147).

Collectively, BCG is capable of stimulating immune responses and triggering signaling molecular pathway in interaction with miRNAs. It seems that BCG instillation can influence miRNAs expression in BC tissue. However, further studies should be conducted to shed more light on this interaction in BC.

Currently, there is little evidence on how miRNAs can alter NETosis process. However, some studies conducted about the interaction between miRNA expression and neutrophil. In this regard, as study showed that neutrophil-associated miR-99b-5p, miR-191-5p, and miR-197-3p transcript levels were remarkably lower in *mycobacterium tuberculosis* (MT) infections. Differentially expression of miRNAs in neutrophils can predominantly effect the signaling pathways leading to cytokine productions. The reduced expression in MT cases could indicate a lack of inhibition on signaling pathways, which may result in elevated production of pro-inflammatory cytokines such as IFN-γ (148).

As was discussed previously, IFN-γ is a crucial cytokine in immune responses to microbial infections. In this regard, a study demonstrated significance of IFN-γ role in NETs function when lung neutrophils of mice infected by *Streptococcus pneumonia, Staphylococcus aureus*, and *Escherichia coli*. Their results revealed that decreased formation of NETs in IFN-γ-deficient mice could result in the increase in S. *pneumonia* bacterial numbers (149, 150).

Gantier comprehensively investigated roles of role of miRNAs in neutrophil's formation, function, and biology and reported a list including 48 miRNAs that are expressed in neutrophils (149). Thus, we found that miRNAs have important role in regulation of neutrophil biology. Among expressed miRNAs in neutrophil, miR1 and miR133 were down-regulated in patients with myeloproliferative disorder. Furthermore, miR223 has been demonstrated to be one of the main miRNAs expressed by human granulocytes (CD15). A study reported an essential role of miR223 in neutrophil differentiation by evaluating miR223-deficient mice (151). Ward et al. indicated that miR-34b, miR-328, miR-483-3p, miR-491-3p, miR-595, and miR-1281 miRNAs were up-regulated when neutrophils treated with GM-CSF. Indeed, GM-CSF treatment led to delay apoptosis and senescence (152). IL-8 as a CXC chemokine ligand has a necessary function in the recruitment of human leukocytes specially neutrophils and also produced by various immune cells such as neutrophils, macrophages, and epithelial cells stimulated with TNF-α. miR17 can directly target IL-8 mRNA, therefore miR17 inhibition could cause a drastically increase in IL-8 production (153). On the contrary, miR155 can increase IL-8 secretion from neutrophils of patients with cystic fibrosis by the suppression of SHIP expression. Elevated miR-155 levels can straightly decrease levels of SHIP-1, which ordinarily destabilize IL-8 mRNA via Akt signaling (154). Hence, therapeutic approaches should focus on decreasing miR155 and increasing miR17 levels which could dampen IL-8 production by neutrophils.

Some studies also determined that neutrophils have capability to polarize and migrate toward center of tumor cells that highly express these chemotactic factors (155, 156). As soon as, the maximum production zone of the chemokine to be reached, the gradient of chemotactic concentration vanishes. Chemotactic stimulus can establish NETs formation when high level of receptor is occupied (89). A study with using intravital microscopy in tumors could observe that neutrophils are able to move to the tumor and form NETs (personal communication). In the tumor microenvironment, these structures have been related often to processes that favor metastasis (37, 157, 158). A study in mice also indicated that NETs facilitate the metastasis capacity of tumor cells favoring their migration (159). However, the extent to which NETs can alter the function of other immune cells in the tumor microenvironment has not been directly demonstrated (160).

There are relatively scarce data about the roles of miRNAs in neutrophil biology in the literature. Different expression of miRNAs in tumor microenvironment could be a useful option to prognosis and detection and treatment various cancers specially BC.

## BCG Optimization for Bladder Cancer

Todays, molecular targeting as a novel therapeutic approach is considered for improving survival and prognosis in patients with BC (161). Various studies have shown that abnormally expressed miRNAs involvement to BC progression by exerting as oncogenes or tumor suppressors. Recent reports have demonstrated that the non-invasive detection of miRNAs from body fluids, including urine and blood of BC patients, can be used to improve prognosis and diagnosis (162, 163). Thereby, the recognition of dysregulated miRNAs to promote clinical applications in BC is really pivotal. Autophagy is contributed in several steps of cancer development, and the collection of evidence associating the dysregulation of autophagy-related miRNAs in cancer has arisen remarkably (164). As yet, about 400 miRNAs have been accredited as predicted to have interactions associated with autophagy (164). In this respect, a study identified various dysregulated miRNAs in BC (165). For instance, the findings of some studies revealed that miR-99a-5p exhibited a tumor suppressor role via targeting mTOR in BC (166). Additionally, miR-30a-5p was another miRNA that increased drug sensitivity to cisplatin by targeting Beclin-1 and ATG5 in BC (167). Thereby, the performed studies have been demonstrated a promising effect of the miRNAs in BC therapy (161). On the other hand, NETs formation in tumor regulation of autophagy by miRNAs can enhance the NETs formation in patients with BC (13, 113, 168). Generally, NETs formation can facilitate BCG performance in BC treatment. Autophagy and NETs are able to suppress tumor activity through specific mechanisms including mTOR signaling pathway and produced ROS of neutrophils, respectively (13, 65). Thus, promoting NETs performance and modifying autophagy by miRNAs can be utilized to improve BCG therapy in patients with BC. Additional efforts are essential to assess the therapeutic roles of candidate miRNAs and its interaction between NETs formation and autophagy pathway. Eventually, further investigations are necessary to further clarify novel RNA networks in BC cells.

## Conclusion

BCG therapy is usually prescribed to the patients with non-muscle invasive BC. Review of the current evidence shows that miRNAs play significant roles in triggering and regulating autophagy process and autophagy-induced NETs formation, which subsequently can promote BCG therapy in patients with BC. Moreover, BCG-induced NETs have been reportedly to exert cytotoxic effects, induce apoptosis, cell cycle arrest, and inhibition of tumor cells migration into bladder environment. Moreover, neutrophils can prim T cells and activate DCs to robust immune responses against tumor growth. Therefore, it could be concluded that utilization of miRNAs network as a therapeutic approach may reinforce BCG function at high efficiency through inducing autophagy which in turn can enhance ROS producing and NETs formation by stimulating neutrophils.

## Author Contributions

CYM and NX were responsible for the conception and design of the study. XX performed the study retrieval. XX and YD contributed to quality evaluation. CYM and XX contributed to the data collection and statistical analysis. CYM drafted the manuscript. NX and CYM were responsible for the revision of the manuscript. The final manuscript had been read and approved by all authors.

## Funding

This work was supported by the Zhejiang Provincial Natural Science Foundation of China (No. LQ19H160027 to CYM).

## Conflict of Interest

The authors declare that the research was conducted in the absence of any commercial or financial relationships that could be construed as a potential conflict of interest.

## Publisher's Note

All claims expressed in this article are solely those of the authors and do not necessarily represent those of their affiliated organizations, or those of the publisher, the editors and the reviewers. Any product that may be evaluated in this article, or claim that may be made by its manufacturer, is not guaranteed or endorsed by the publisher.
